# Functional expression and pharmaceutical efficacy of cardiac-specific ion channels in human embryonic stem cell-derived cardiomyocytes

**DOI:** 10.1038/s41598-017-14198-y

**Published:** 2017-10-23

**Authors:** Han Sol Kim, Jung Won Yoon, Hongliang Li, Geun Ok Jeong, Jin Ju Park, Sung Eun Shin, Il Ho Jang, Jae Ho Kim, Won Sun Park

**Affiliations:** 1 Department of Physiology, Kangwon National University School of Medicine, Chuncheon, 24341 Republic of Korea; 2 Department of Physiology, School of Medicine, Pusan National University, Yangsan, 50612 Republic of Korea; 3 Department of Oral Biochemistry and Molecular Biology, Pusan National University School of Dentistry, Yangsan, 50612 Republic of Korea; 4 Research Institute of Convergence Biomedical Science and Technology, Pusan National University Yangsan Hospital, Yangsan, 50612 Republic of Korea

## Abstract

Cardiomyocytes differentiated from human pluripotent stem cells provide promising tools for screening of cardiotoxic drugs. For evaluation of human pluripotent stem cell-derived cardiomyocytes for cardiotoxicity test, in the present study, human embryonic stem cells (hESCs) were differentiated to cardiomyocytes, followed by metabolic selection to enrich the differentiated cardiomyocytes. The highly purified hESC-derived cardiomyocytes (hESC-CMs) expressed several cardiomyocyte-specific markers including cTnT, MLC2a, and α-SA, but not pluripotency markers, such as OCT4 and NANOG. Patch clamp technique and RT-PCR revealed the expression of cardiomyocyte-specific Na^+^, Ca^2+^, and K^+^ channels and cardiac action potential in hESC-CMs. To explore the potential use of hESC-CMs as functional cardiomyocytes for drug discovery and cardiotoxicity screening, we examined the effects of bisindolylmaleimide (BIM) (I), which inhibits native cardiac Ca^2+^ channels, on the Ca^2+^ channel activity of hESC-CMs. We observed a similar response for the BIM (I)-induced modulation of Ca^2+^ channels between hESC-CMs and native cardiomyocytes through *L*-type Ca^2+^ channel current. These results suggest that hESC-CMs can be useful for evaluation of pharmaceutical efficacy and safety of novel drug candidate in cardiac research.

## Introduction

Cardiovascular disease is a leading cause of mortality and morbidity, and chronic cardiovascular diseases results in major public health burdens. Cardiomyocyte research is important to understand the causes, treatment and prevention of cardiovascular diseases; however, such research poses several critical challenges. Postnatal cardiomyocytes have little or no regenerative capacity^1^, and obtaining human cardiomyocytes in amounts sufficient for research is difficult. In addition, some drugs for cardiovascular diseases carry a risk of damage to the cardiomyocytes, producing abnormal cardiac function, and emphasizing the need for safety assessment of such drugs in clinical trials. Human embryonic stem cell-derived cardiomyocytes (hESC-CMs) are efficient strategies in cardiac research, regenerative therapy, disease modeling, and cardiomyocyte-based models for cardiotoxicity and drug discovery.

To generate hESC-CMs, various research groups developed the methods for cardiomyocyte differentiation and characterized their functional properties^2^. Since the first attempt in human pluripotent stem cells in 1998, different methods have been proposed including co-culture system, 3D culture, and addition of morphogens (Activin A and BMP4)^3^. hESC-CMs provide a significant advantage over animal models due to the physiological differences, including beating rate, ion channel expression, and drug toxicity. Besides hESC-CMs exhibit mostly ventricular-like cardiomyocytes, they have been reported to be relatively immature^4^. In the current study, to generate hESC-CMs for application of drug screening, we adopted hESC-CMs differentiation method utilizing small molecules that modulate the Wnt signaling pathway, in which the differentiated cardiomyocytes showed the high expression of cardiac specific markers (cTnT, α-actinin, MLC2a, and MLC2v) and function (action potential)^1^.

Although there are several cardiac markers to identify successful differentiation of stem cells into cardiomyocytes, specific ion channels expressed in cardiomyocyte can be used as well for this purpose. Cardiomyocytes express tetrodotoxin-insensitive Na^+^ channels, L- and T-type Ca^2+^ channels (I_Ca_), transient outward K^+^ (I_to_) channels, ultra-rapid delayed rectifier K^+^ (I_Kur_) channels, rapid delayed rectifier K^+^ (I_Kr_) channels, slow delayed rectifier K^+^ (I_Ks_) channels, inward rectifier K^+^ (I_K1_) channels, and ATP-sensitive K^+^ (K_ATP_) channels^5,6^. Overall, ion channels in cardiomyocytes contribute to the generation of action potential shape. Therefore, the detection of functional ion channels and action potential is essential to prove successful differentiation of stem cells into cardiomyocytes.

In this study, we explored the differentiation of human embryonic stem cells (hESCs) into cardiomyocytes by detecting cardiac marker proteins and recording cardiac specific ion channels. Furthermore, to prove the pharmaceutical efficacy of hESC-CMs in cardiac research, we investigate the effect of bisindolylmaleimide (BIM) (I), which inhibits native cardiac Ca^2+^ channels^7^, on Ca^2+^ channels expressed in hESC-CMs.

## Results

### Phenotypic characterization of cardiomyocytes differentiated from hESCs

**Figure 1:**
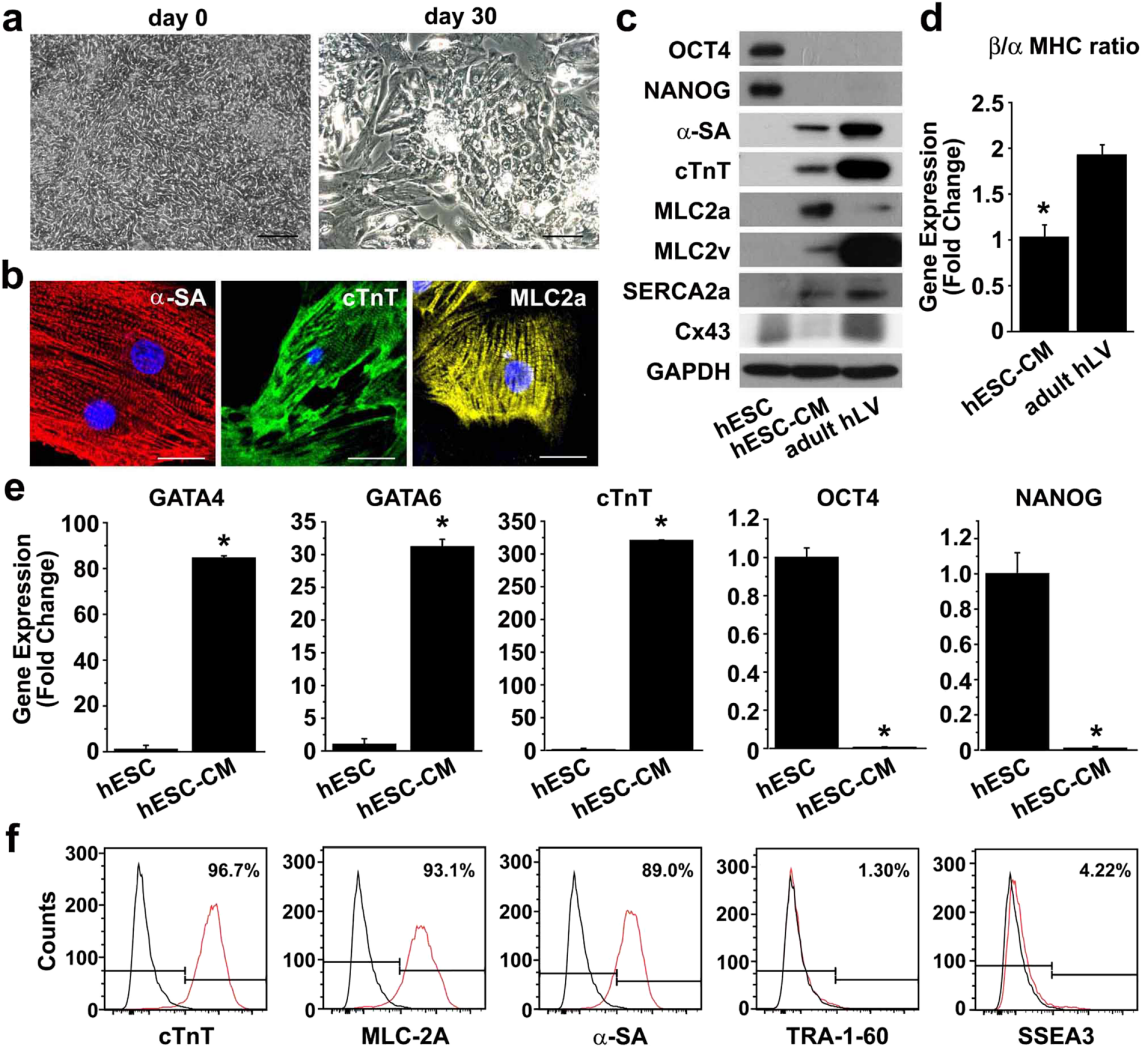
Characterization of cardiomyocytes differentiated from H9 hESCs. (**a**) Bright field images of H9 cells at day 0 (left panel) and differentiating cells at day 30 (right panel). Scale bar = 100 μm (**b**) Differentiated cardiomyocytes (hESC-CMs) at day 30 were subjected to immunofluorescent staining with antibodies against α-SA (red), cTnT (green), or MLC2a (yellow). Nuclei were stained with DAPI, and the merged images are shown. Scale bar = 20 μm. (**c**) Protein expression in undifferentiated H9 cells, hESC-CMs, and adult human left ventricular tissue (adult hLV) was assessed by Western blot analysis with antibodies against pluripotency markers (OCT4 and NANOG) and cardiomyocyte markers (α-SA, cTnT, MLC2a, MLC2v, SERCA2a and Cx43). (**d**) The mRNA levels of α-MHC and β-MHC in hESC-CMs and adult hLV were accessed by quantitative RT-PCR and the ratio of β-MHC/α-MHC mRNA levels was determined (*n* = 3). *P < 0.05. (**e**) Gene expression in hESC-CMs at day 30 and hESCs was accessed by quantitative RT-PCR with cardiomyocyte markers (GATA4, GATA6, and cTnT) and pluripotency markers (OCT4 and NANOG) *n* = 3. *P < 0.05. (**f**) Flow cytometry analysis of hESC-CMs at day 30 with antibodies against cardiomyocyte markers (cTnT, MLC2a, and α-SA) and pluripotency markers (TRA-1-60 and SSEA3) is shown.

Prior to cardiomyocyte differentiation, pluripotent hESCs were clearly defined with a tight pack of brightly-clean, round cells (Supplementary Fig. S1a). hESC colonies expressed SOX2, OCT4, TRA-1–81, TRA-1-60 and SSEA-4, pluripotent stem cell markers, but not cardiomyocyte differentiation marker cTnT (Supplementary Fig. S1b and c). hESCs were differentiated to cardiomyocytes and purified with the protocol summarized in Supplementary Fig. S2. At day 20, the cells exhibited a mixture of poorly-differentiated cells and well-differentiated cardiomyocytes, demonstrating morphology transition from hESCs to cardiomyocytes (Fig. 1a). To enrich well-differentiated cardiomyocytes, the differentiated cardiomyocytes were exposed to glucose-depleted lactate medium. After selection with lactate medium, spontaneous and autonomous beating of hESC-CMs was observed (Supplementary Movie S1) and cardiac enrichment was verified by flow cytometry analysis. Immunofluorescent staining showed that the differentiated cells at day 30 extensively expressed α-SA, cTnT, and MLC2a with organized sarcomere structure (Fig. 1b). Western blot analysis showed expression of the cardiomyocyte-specific markers, including α-SA, cTnT, MLC2a, and MLC2v, while OCT4 and NANOG expression decreased considerably (Fig. 1c). To further confirm the cardiomyocyte-specific phenotypes of the hESC-CMs, gene expression was evaluated by qRT-PCR. The expression of cardiomyocyte-specific markers, such as GATA4, GATA6, and cTnT, increased markedly, whereas the expression of pluripotency markers, such as OCT and NANOG, decreased significantly (Fig. 1e). In addition, flow cytometry analysis showed that the majority of cells expressed cardiomyocyte markers, such as of cTnT, MLC2a, and α-SA, but not pluripotency markers, such as TRA-1-60 and SSEA3 (Fig. 1f). However, the expression levels of α-SA and cTnT in hESC-CMs were markedly lower than those in adult human left ventricle tissues (adult hLV) (Fig. 1c). Moreover, the expression levels of Cx43, a component of cardiomyocyte gap junction, and SERCA2a, a calcium ATPase related to cardiomyocyte function^8,9^, were substantially lower in hESC-CMs than that in adult hLV. To further confirm the immaturity of hESC-CMs, we quantified the ratio of myosin heavy chain (MHC) isoforms, a well-known cardiomyocyte-specific marker, by using the qRT-PCR. In human hearts, there are more β-MHC than α-MHC at all stages; however, it has been reported that there is more α-MHC in fetal than adult hearts^10,11^. The hESC-CMs exhibited relatively lower ratio of β-MHC/α-MHC expression than adult hLV (Fig. 1d). These data suggest that hESCs can be differentiated into cardiomyocytes *in vitro*, but the hESC-CMs exhibit immature phenotypes.

### Characterization of Na^+^ channel activities in hESC-CMs

**Figure 2:**
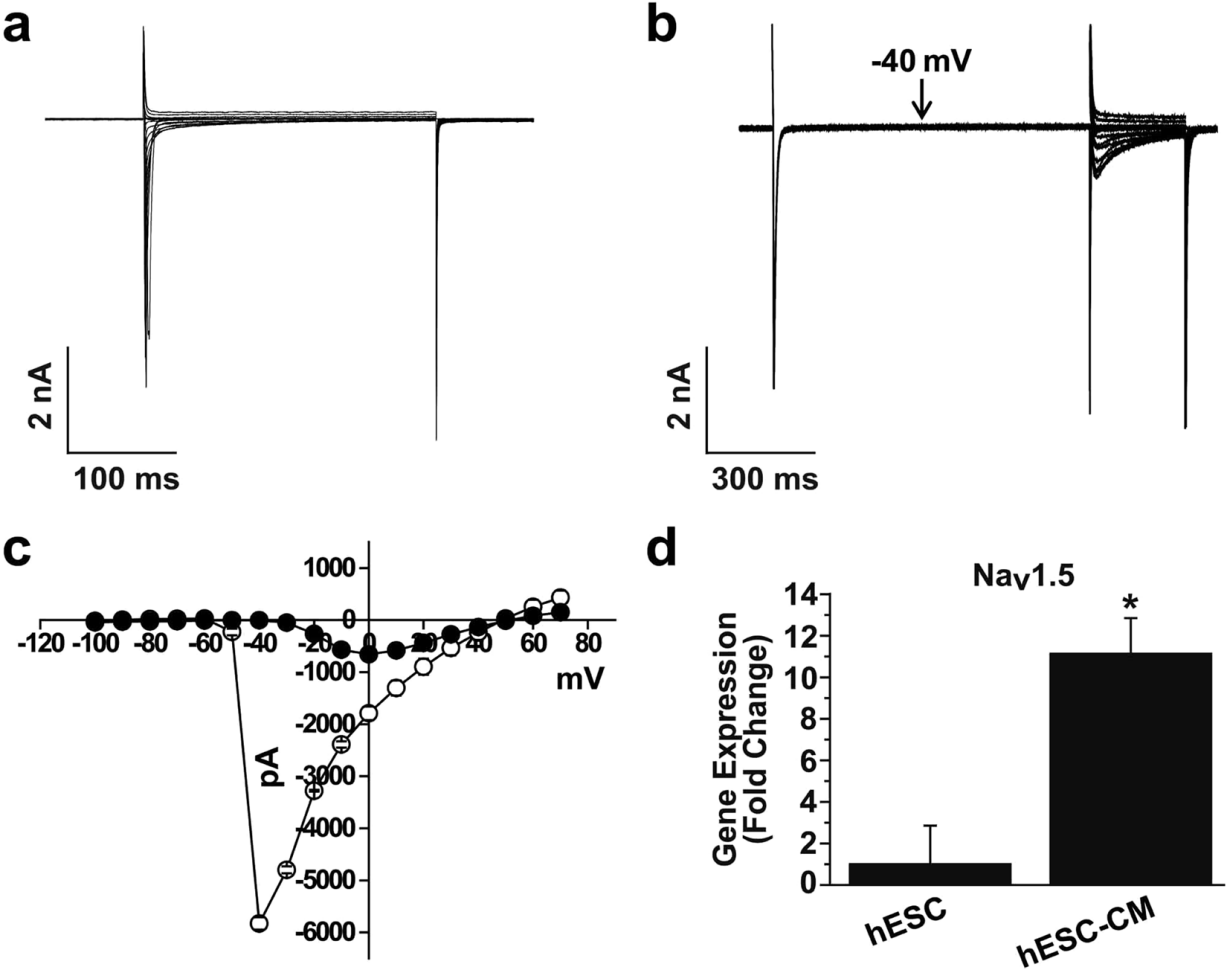
Electrophysiological recordings of Na^+^ channels in hESC-CMs. (**a**) Superimposed current traces were evoked by 300-ms depolarizing step pulses from −100 to +70 mV from holding potential of −120 mV. (**b**) Representative current traces were elicited by the first step depolarizing pulses as described in (A), then returning to −40 mV for 1 s. The second identical step depolarizing pulses were applied. (**c**) The current-voltage (*I*-*V*) relationship for the panel (**a**, open circle) and (**b**, closed circle). *n* = 7. (**d**) Expression of Nav1.5 subtype in H9 cells and hESC-CMs was accessed by quantitative RT-PCR. *n* = 5. *P < 0.05.

Voltage-gated Na^+^ channels are typical of cardiomyocytes. Among nine subtypes, Tetrodotoxin-insensitive subtypes, such as Nav1.5 and Nav1.8, have been identified in cardiomyocytes^6^. To record the Na^+^ current, electrical activities of K^+^ channels were inhibited by inclusion of Cs^+^ in the external and internal solutions. Na^+^ currents were elicited by step depolarizing pulses from −100 to +70 mV in steps of 10 mV with holding potential of −120 mV. As shown in Fig. 2a, Na^+^ currents were rapidly activated and then inactivated after reaching the peak. Maximal peak currents were observed in voltage of −40 mV (Fig. 2c, open circle), suggesting that the recorded currents were Na^+^ currents. To confirm the Na^+^ current, we recorded Na^+^ current using similar pulse protocols, then returned potential at −40 mV for 1 s to inactivate Na^+^ current. Figure 2b and c (closed circle) showed that the most Na^+^ currents induced by second steps pulses were inhibited. To further prove the molecular identity of the recorded Na^+^ currents, we compared the expression levels of Nav1.5 subtype between hESCs and hESC-CMs. As shown in Fig. 2d, the expression level of Nav1.5 subtype was greater in hESC-CMs than hESCs, supporting functional expression of Na^+^ channels in hESC-CMs.

### Characterization of Ca^2+^ channel activities in hESC-CMs

**Figure 3:**
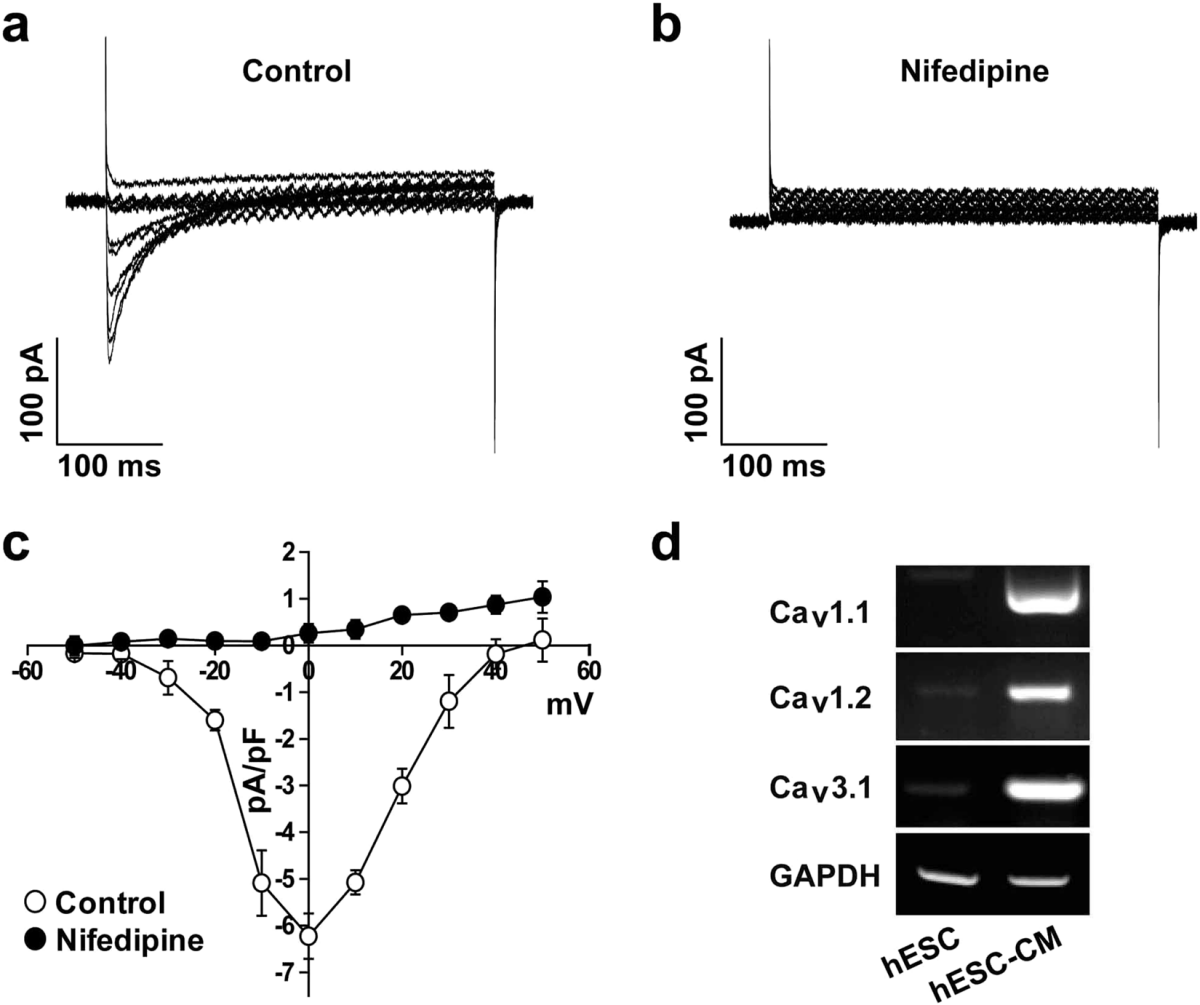
Electrophysiological recordings of Ca^2+^ channels in hESC-CMs. Superimposed current traces were recorded by applying 500-ms step depolarizing pulses in the voltage range of −50 to +50 mV from a holding potential of −50 mV under control condition (**a**) and in the presence of 10 µM Nifedipine (**b**). (**c**) *I*-*V* relationship of peak current in the absence (open circle) and presence of Nifedipine (closed circle). *n* = 6. (**d**) Expression of Cav1.1, Cav1.2, and Cav3.1 subtypes in H9 cells and hESC-CMs was accessed by RT-PCR. *n* = 5. *P < 0.05.

To record the *L*-type Ca^2+^ current, electrical activities of other K^+^ channels were reduced by addition of Cs^+^ in the external and internal solutions, as similar to Na^+^ current recordings. Furthermore, holding potential was maintained at −50 mV to inhibit Na^+^ and *T*-type Ca^2+^ channels. *L*-type Ca^2+^ currents were evoked by applying step depolarizing pulses from −50 mV to +50 mV in steps of 10 mV at −50 mV holding potential. As shown in Fig. 3a, voltage-dependent inward currents were recorded. These currents have maximal peak current at voltage of 0 mV (Fig. 3c). To verify that recorded currents were *L*-type Ca^2+^ currents, we applied *L*-type Ca^2+^ channel inhibitor, Nifedipine. Treatment of hESC-CMs with 10 µM Nifedipine effectively inhibited the recorded current throughout the whole voltage range, which suggested that the recorded currents were *L*-type Ca^2+^ current (Fig. 3b and c). We also investigate the molecular subtypes of Ca^2+^ channels. As shown in Fig. 3d, the expression levels of *L*-type Ca^2+^ channel subtypes (Cav1.1 and Cav1.2) and *T*-type Ca^2+^ channel subtype (Cav3.1) were highly increased in hESC-CMs. It has been reported that Cav1.2 and Cav3.1 are associated with cardiac muscle^12–14^. These results confirm the functional expression of Ca^2+^ channels in the hESC-CMs.

### Characterization of K^+^ channel activities in hESC-CMs

**Figure 4:**
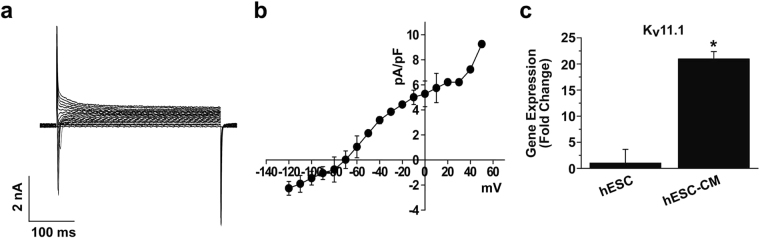
Electrophysiological recordings of K^+^ channels in hESC-CMs. (**a**) Whole-cell K^+^ currents of hESC-CMs. (**b**) Summary of *I*-*V* relationship of panel (a). *n* = 5. (**c**) Expression of Kv11.1 subtype in H9 cells and hESC-CMs was accessed by quantitative RT-PCR. *n* = 5. *P < 0.05.

To record the general K^+^ current, we simply applied step depolarizing pulses (between −120 and +40 mV from holding potential of −70 mV) with the *L*-type Ca^2+^ channel inhibitor Nifedipine (10 µM). As shown in Fig. 4a and b, shapes similar to native cardiac K^+^ currents were observed. However, I_K1_ currents were not detectable in our recordings. We investigate the expression of representative cardiac molecular subtype, Kv11.1 (I_Kr_) in hESCs and hESC-CMs. Figure 4c shows that the expression level of Kv11.1 was up-regulated in hESC-CMs compared with undifferentiated hESCs. These results suggest that hESC-CMs express cardiac K+ channels, although I_K1_ currents are deficient compared with native cardiomyocytes.

### Recordings of action potential in hESC-CMs

**Figure 5:**
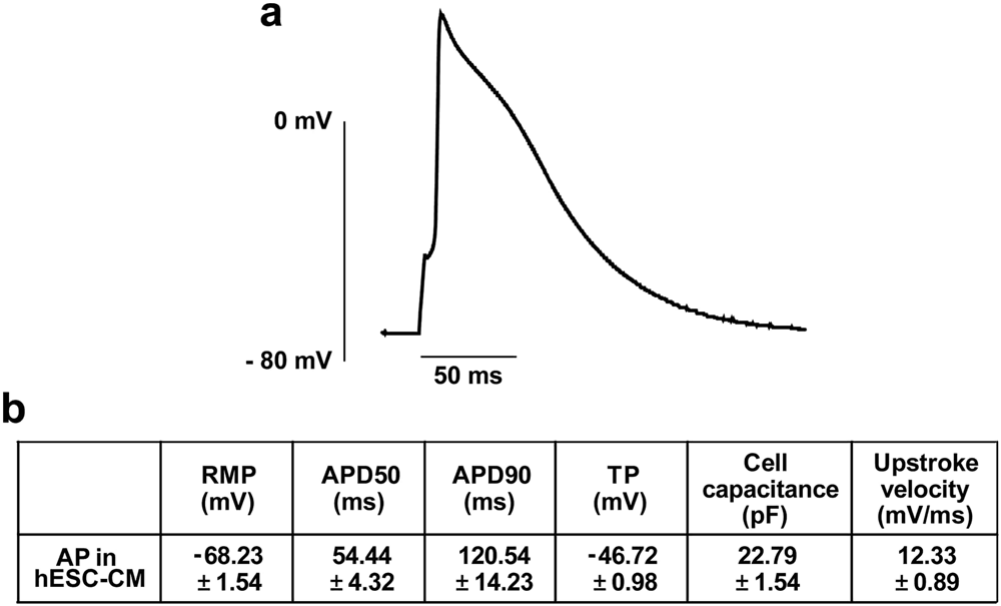
Action potential in hESC-CMs. Action potential was induced by minimal current injection to overcome threshold in current clamp mode. (**a**) Representative trace of action potential recorded in hESC-CMs. (**b**) Analysis of action potential. APD_50_ and APD_90_ means action potential durations at 50% and 90% of repolarization; RMP, resting membrane potential; TP, threshold potential. *n* = 7.

In order to investigate the electrical activity of differentiated cardiomyocytes, whole-cell patch clamp technique was performed. Figure 5a shows representative traces of spontaneous action potential recorded from hESC-CMs. The analysis of action potential such as resting membrane potential, action potential duration, cell capacitance, threshold potential, and upstroke velocity was described in Fig. 5b. Although the shape of action potential did not completely correspond to the native cardiac cells, the shape is similar to the shape of action potential when blocking I_K1_ channels in the native cardiomyocytes. These results are consistent with the patch clamp result that I_K1_ currents were not detectable.

### BIM (I)-induced inhibition of the L-type Ca^2+^ currents on hESC-CMs

**Figure 6:**
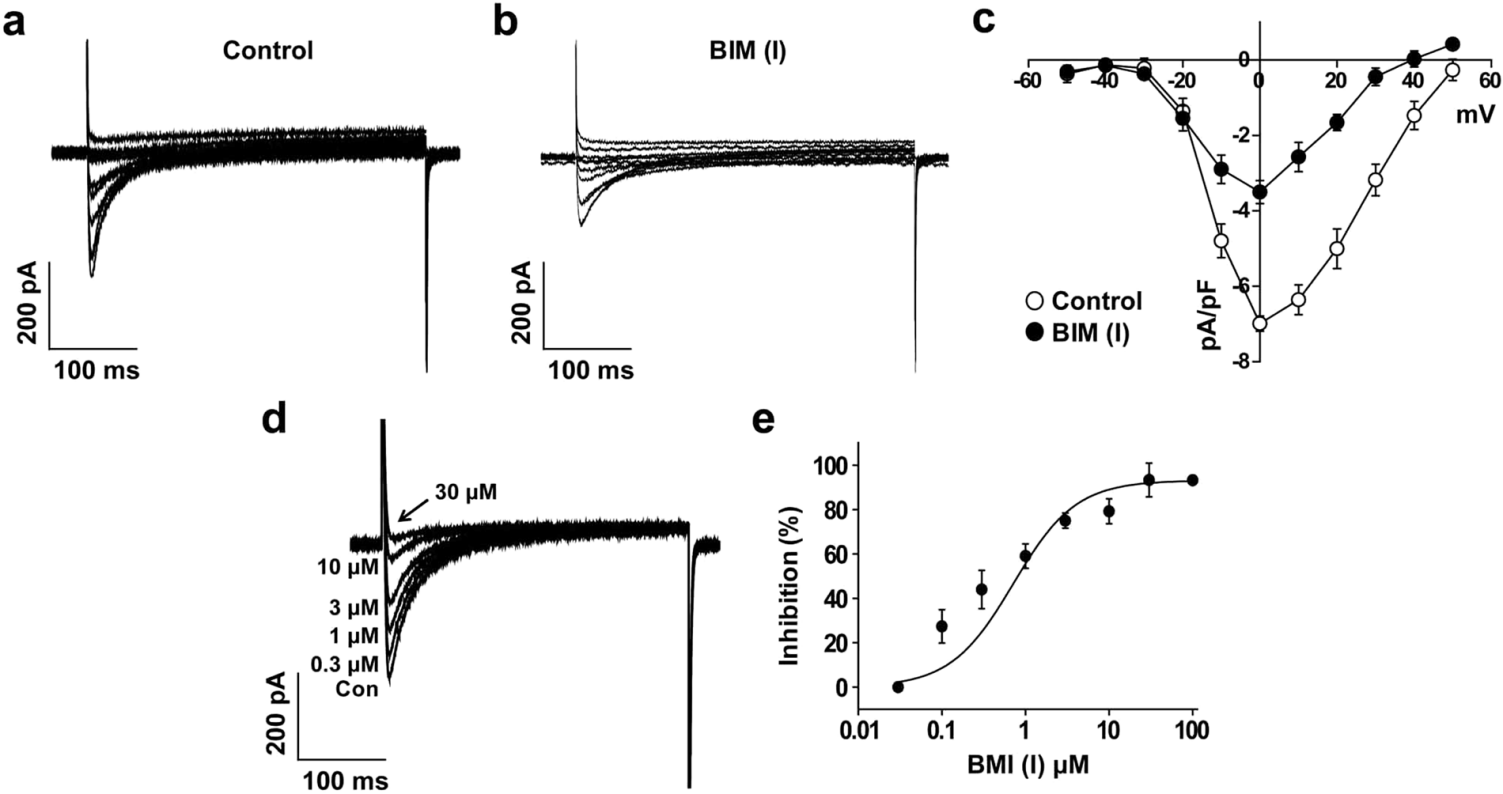
The effect of BIM (I) on *L*-type Ca^2+^ current in hESC-CMs. Superimposed current traces under control conditions (**a**) and in the presence of BIM (I) (1 μM) (**b**) were obtained by applying the same pulse protocol for Fig. 3. (**c**) *I-V* relationship at the peak Ca^2+^ current in the absence (open circle) and presence of BIM (I) (1 µM) (closed circle). *n* = 6. *P < 0.05. (**d**) Superimposed current traces were recorded by applying one-step depolarization pulse of 0 mV from a holding potential of −50 mV in the presence of BIM (I) (0, 0.03, 0.1, 0.3, 1, 3, 10, 30, and 100 μM). (**e**) BIM (I)-induced inhibition of the *L*-type Ca^2+^ current was measured at peak current, and normalized to the current amplitude observed in the absence of BIM (I). Normalized currents were fitted with the Hill equation. *n* = 6.

To explore whether hESC-CMs can be used for drug screening and cardiotoxicity test, we examined the effect of BIM (I), a specific inhibitor of protein kinase C (PKC), on *L*-type Ca^2+^ currents in hESC-CMs, since our previous report suggested that BIM (I) inhibited the *L*-type Ca^2+^ currents expressed in native cardiac (ventricular) myocytes^7^. Application of BIM (I) (1 μM) rapidly reduced the *L*-type Ca^2+^ currents within 1 min (Fig. 6a and b). At 0 mV, the current density of *L*-type Ca^2+^ channel was −6.99 ± 0.20 pA/pF under control conditions and −3.50 ± 0.30 pA/pF in the presence of BIM (I) (1 μM) (Fig. 6c). Although the current density of *L*-type Ca^2+^ current in hESC-CMs was smaller than that of native cardiac cells, the inhibition ratio of *L*-type Ca^2+^ channel by BIM (I) was more sensitive in hESC-CMs (50% inhibition by 1 μM BIM (I)) compared with native cardiomyocytes (47% inhibition by 3 μM BIM (I))^7^.

To test the concentration-dependent inhibition of BIM (I) on the *L*-type Ca^2+^ currents, various concentrations of BIM (I) (0.03, 0.1, 0.3, 1, 3, 10, 30, or 100 μM) were applied before measurement of *L*-type Ca^2+^ currents. Currents were elicited by one-step depolarizing pulses to 0 mV from a holding potential of −50 mV. As shown in Fig. 6d, *L*-type Ca^2+^ currents were inhibited by treatment with increasing doses of BIM (I). The dose-response curve was obtained by nonlinear least-squares fit of the Hill equation to the concentration-inhibition data with an IC_50_ value of 0.71 ± 0.20 μM and a Hill coefficient (n) of 1.16 ± 0.22 (Fig. 6e). These data suggest that BIM (I) inhibited *L*-type Ca^2+^ channel of hESC-CMs in a concentration-dependent manner.

### Effect of other PKC inhibitors on L-type Ca^2+^ channel in hESC-CMs

**Figure 7:**
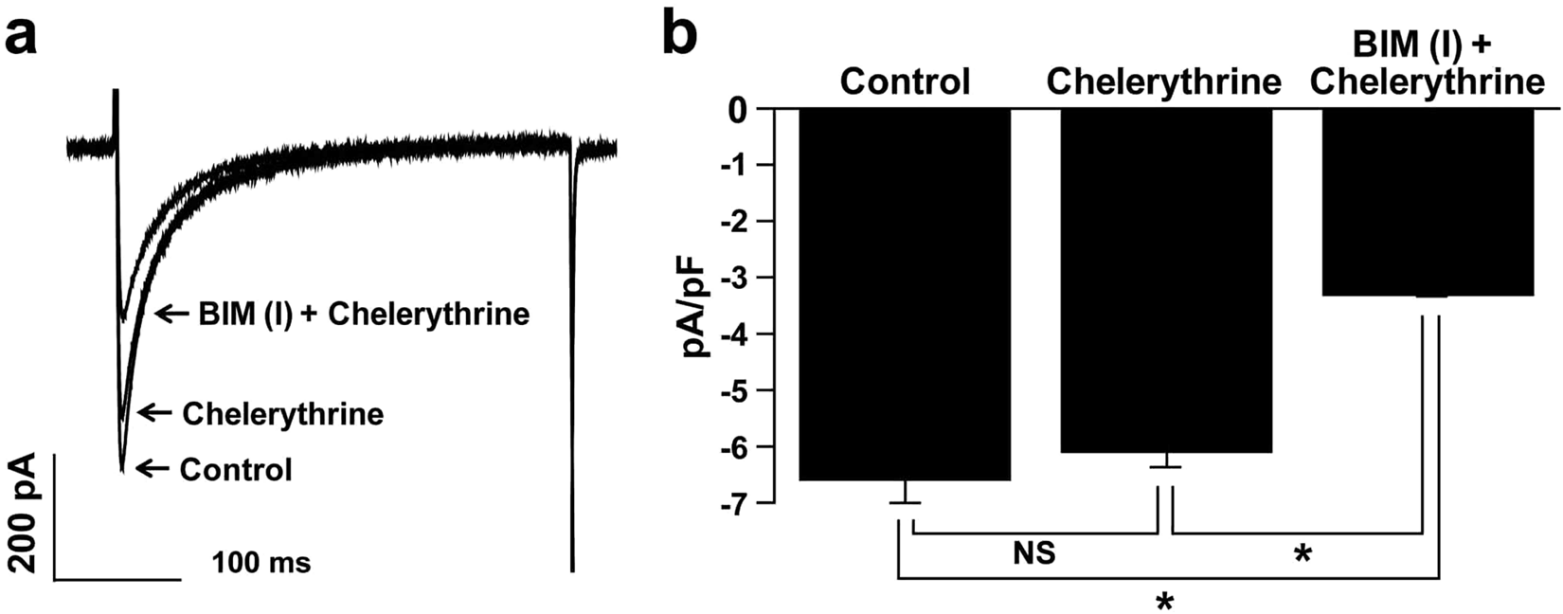
The effects of another PKC inhibitor, chelerythrine, on the BIM (I)-induced inhibition of *L*-type Ca^2+^ currents. (**a**) Superimposed current traces in control conditions, in the presence of Chelerythrine, and in the presence of Chelerythrine + BIM (I). (**b**) Summarized data from traces in (**a**). *n* = 6. *P < 0.05. NS = not significant.

To demonstrate whether BIM (I)-induced inhibition of *L*-type Ca^2+^ channels was due to the inhibition of PKC, we tested the effect of another PKC inhibitor, Chelerythrine on *L*-type Ca^2+^ channel. As shown in Fig. 7a, application of Chelerythrine (3 μM) did not affect the *L*-type Ca^2+^ current and did not alter the BIM (I)-induced inhibition of *L*-type Ca^2+^ channel. The *L*-type Ca^2+^ channel current densities were −6.59 ± 0.41 pA/pF in control conditions, −6.09 ± 0.28 pA/pF in the presence of Chelerythrine, and −3.31 ± 0.03 pA/pF in the presence of Chelerythrine together with BIM (I) (1 μM) (Fig. 7b). These results suggest that the inhibitory effect of BIM (I) on *L*-type Ca^2+^ channel is not due to inhibiting PKC activity.

## Discussion

Ion channels are critical for all aspects of cardiac functions, including rhythmicity and contractility, by regulating cardiac action potential. The action potential shape and duration are determined by the sum of inward and outward currents including I_Na_ current, I_to_ current, I_Kur_ current, I_Kr_ current, I_Ks_ current, I_K1_ current, and I_Ca_ current^6,15–17^. In the present study, we demonstrated differentiation of hESCs into functional cardiomyocytes expressing cardiomyocyte-specific markers and ion channels. The hESC-CMs expressed functional ion channels selective for Ca^2+^, Na^+^, and K^+^ ions. The results of the current study may have important implications for the potential applications of the hESC-CMs for drug screening that modulate ion channels.

L-type Ca^2+^ channels are key ion channels implicated in cardiac physiology and pharmacology, and largely responsible for the action potential plateau^17^. Whole-cell Ca^2+^ transients in pluripotent stem cell-derived cardiomyocytes depend on both Ca^2+^ influx via L-type Ca^2+^ channels and intracellular Ca^2+^ store release, as previously documented in mouse^18,19^ and hESC-CMs^20,21^. The importance of the L-type Ca^2+^ current in generating whole-cell Ca^2+^ transients in these cells was manifested by the elimination of these transients in the absence of external Ca^2+^ or in the presence of Nifedipine, a selective L-type Ca^2+^ channel antagonist. A similar requirement for external Ca^2+^ and the consequent trans-sarcolemmal Ca^2+^ influx was documented in adult cardiomyocytes^22–24^, hESC-CMs^21^ and mouse ESC-CMs^25,26^. We have previously reported that BIM (I), a PKC inhibitor, inhibited *L*-type Ca^2+^ channels in rat native ventricular cells with IC_50_ value of 3.31 µM^7^. In this study, we obtained the IC_50_ value of 0.71 µM, therefore, it is likely that the inhibitory effect of BIM (I) on *L*-type Ca^2+^ channels in hESC-CMs is more sensitive than that of rat ventricular cells. Although the reason for the higher sensitivity of hESC-CMs to BIM (I) is not clear yet, these results suggest that hESC-CMs can be useful for drug screening based on Ca^2+^ channel.

Based on the action potential phenotypes recorded in isolated cells, differentiated CMs have been reported to have a heterogeneous population classified as atrial-, nodal-, or ventricular-like^27^. Cell culture conditions and differentiation protocols may influence these action potential properties^28,29^. In our results, the action potential recorded in hESC-CMs has a short action potential duration compared with native cardiomyocytes. Moreover, we showed that the *L*-type Ca^2+^ channel current recorded in hESC-CMs was smaller than native cardiomyocytes. Ca^2+^ current is a major inward current maintaining the plateau phase of action potential in native cardiomyocytes, and calcium influx results in cytoplasmic enrichment of Ca^2+^ and ventricular myocyte contraction^23^. Therefore, inhibition of Ca^2+^ current shortens action potential duration and increases the heart rate. The shortening of action potential duration by reducing Ca^2+^ current amplitude is commonly observed in neonatal cardiomyocytes. The action potential duration and Ca^2+^ current amplitude of neonatal cardiomyocytes were shorter and smaller than those of adult in guinea-pig ventricular myocytes^26^. In addition, Ca^2+^ current amplitude of neonatal cardiomyocytes was small compared with adult cardiomyocytes in rabbit and murine models^26,30^. Therefore, it is likely that hESC-CMs used in our experiment possess Ca^2+^ channel characteristics similar to neonatal cardiomyocytes.

Several reports have suggested that hESC-CMs have relatively longer action potential duration than that of our recordings^31–33^. Although we could not address the detail reasons for the relative short action potential duration compared to the results of other study groups, it may be caused by the small Ca^2+^ current amplitude. In fact, Ca^2+^ current recorded in the present study is half of the Ca^2+^ current that reported in previous papers. For this reason, action potential duration recorded in our group is relatively short compared to other papers (Fig. 5b). Regarding the resting membrane potential, various results were elicited differently for each group. Some studies have reported that the resting membrane potential remains stable at −60 ~ −70 mV, however, the other studies have reported that the resting membrane potential was maintained at −40 ~ −60 mV, which elicits spontaneous action potential. It is difficult to explain the maintenance of resting membrane potential at −60 ~ −70 mV even though I_K1_ is not present. However, other ion channels, such as K_ATP_ and SK_Ca_ channel, are likely to play an important role in maintaining stable resting membrane potential. This will need to be clarified through further research.

Several families of voltage-gated potassium channels are expressed in cardiomyocytes and together provide the majority of the outward current responsible for action potential repolarization^6^. I_K1_ currents expressed in cardiomyocytes are important in maintaining and stabilizing resting membrane potential^34,35^. Furthermore, The I_K1_ currents were assigned for shaping the initial depolarization, final repolarization, and resting phases of the ventricular action potential^30,35,36^. In our recordings, however, I_K1_ currents were not detectable (Fig. 4) and low I_k1_ currents may be another reason for shorting of action potential duration. Consistently, it has been demonstrated that the density of I_K1_ is either absent or significantly smaller in differentiated CMs than that reported for human native ventricular CMs^27^. Since previous reports suggested that action potential duration was reduced in embryonic and neonatal stages compared with the adult stage, and I_K1_ currents were crucial in the generation and shape of action potential^37,38^. These results support the notion that the hESC-CMs used in our experiment could be more similar with the cardiomyocytes from embryonic or neonatal stages.

BIM (I), a specific PKC inhibitor, has known to decrease L-type Ca^2+^ channels activity by altering the voltage-sensitivity of channels. In fact, BIM (I) shifted the steady-state inactivation curve toward a more negative potential suggesting that BIM (I) could directly interact with *L*-type Ca^2+^ channels^7^. Considering that *L*-type Ca^2+^ channels play an important role in cardiac function by regulating excitation-contraction coupling, action potential duration, and intracellular Ca^2+^-dependent signaling pathways, application of BIM (I) reduces cardiac contractility, action potential duration, and PKC-dependent signaling pathways. In the present study, we applied BIM (I) on the Ca^2+^ channels expressed in hESC-CMs to evaluate pharmaceutical efficacy in cardiac research. As similar results with native cardiac Ca^2+^ channels, BIM (I) efficiently reduced the Ca^2+^ channel expressed in hESC-CMs suggesting that hESC-CMs will be useful for cardiac functional studies, specifically Ca^2+^ channels.

Overall, the human pluripotent stem cell-derived CMs present a new and rapidly developing technology with exciting applications, and with further refinements it could pave the way for the development of personalized medicine for cardiovascular diseases^27^. Although the hESC-CMs have several barriers to direct use in clinical field, but hESC-CMs used in this experiment are valuable in the field of cardiac research, specifically cardiotoxicity and the development of new pharmaceuticals. To resolve the problems that the hESC-CMs exhibit the phenotypes of neonatal cardiomyocytes, further experiments are necessary to develop technologies for differentiation of human pluripotent stem cells into functionally matured cardiomyocytes.

## Materials and Methods

### Materials

DMEM/F12, glucose-free DMEM, RPMI1640, FBS, Penicillin-streptomycin, Collagenase type IV, Dispase, Accutase, B27 supplement, B27 supplement minus insulin, and 0.05% trypsin-EDTA were purchased from Thermo Fisher Scientific (Waltham, MA). mTeSR1 and mTeSR1 5 × supplement were purchased from STEMCELL Technologies Inc. (Vancouver, Canada). Humans ES cell line, H9 (WA09), were purchased from the WiCell Research Institute (Madison, WI). Matrigel (#354277) was purchased from Corning Life Sciences (Tewksbury, MA). Antibodies against TRA-1-60 (#560884), TRA-1-81 (#560883), SSEA4 (#561565), SSEA3 (#560881) were purchased from BD Biosciences (San Jose, CA). Antibodies against Oct4 (#AB181557) and Sox2 (#AB59776) were purchased from Abcam (Cambridge, United Kingdom). Anti-GAPDH antibody (#MAB374) was purchased from EMD Millipore (Billerica, MA). Anti-cTnT antibody (#130-106-746, Miltenyi Biotec BmbH, Bergisch Gladbach, Germany), anti-MLC2a antibody (#311011, Synaptic Systems GmbH, Goettingen, Germany), and anti-MLC2v antibody (#3671, Cell Signaling Technology, Danvers, MA) were purchased from the indicated companies. Mitomycin C, Gelatin, Y27632, anti-α-SA antibody (#A7732), and all other unlisted reagents were purchased from Sigma-Aldrich (St. Louis, MO). Adult human left ventricle tissues were obtained as surgical waste from patients undergoing heart transplant operations performed at Pusan National University Yangsan Hospital (Yangsan, South Korea). Written informed consent was obtained from donors and the protocol was approved by the Institutional Review Board of Pusan National University Hospital.

### Culture of human ES cells

Mouse embryonic fibroblasts (MEFs) were purchased from the CEFO Corporation (www.cefobio.com, Seoul, Korea). For preparation of feeder, MEFs on passage 5 were treated with 5 ng/ml Mitomycin C for 1.5 h and seeded to 4 × 10^5^ cells/60 mm dish (0.1% gelatin coated) in DMEM containing 10% FBS and penicillin-streptomycin (125 U/ml). Undifferentiated H9 hESC colonies were cultured on Mitomycin C-treated un-mitogenic MEFs feeder layer. H9 hESC colonies were maintained in DMEM/F12 medium containing 1 × L-glutamax, penicillin-streptomycin (125 U/ml), β-mercaptoethanol (60 μM), 1 × NEAA (#11140, Thermo Fisher Scientific), 20% KSR medium (#10828, Thermo Fisher Scientific) and bFGF (5 ng/ml) (#100-18B, PeproTech, Rocky Hill, NJ) with daily change of media for 4 days. On the 5^th^ day, H9 hESC colonies were passaged by treatment with dissociation medium (DMEM/F12 containing collagenase type IV(200 μg/ml) and Dispase (1 mg/ml)) for 12 min. After dissociation medium treatment, the colonies were harvested by sedimentation and washed with the maintenance medium twice. The collected hESC colonies were cut into pieces of 50~80 μm in diameter by pipetting and plated onto the MEF feeders with 1:3 or 1:4 split ratio, and then incubated at 37 °C under 5% CO_2_. The study protocol was approved by the Public Institutional Bioethics Committee designated by the MOHW. All experiments were performed in accordance with relevant guidelines and regulations.

### Differentiation of hESCs into cardiomyocytes

To differentiate H9 hESCs into cardiomyocytes, hESCs were dissociated to single cells by treatment with Accutase at 37 °C for 7-8 min. Single cell suspension was seeded on Matrigel-coated 6-well culture plate prepared as described^1^. In brief, dissociated cells were plated in mTeSR1 medium supplemented with Y27632 (5 μM) with a density of 1.4 × 10^6^ cells/well, followed by changing to fresh medium every day for 3 days with 80–90% confluence. To initiate differentiation, the media was changed to RPMI/B27 minus insulin media containing the GSK3-specific inhibitor CHIR99021 (14 μM; Selleck Chemicals, Houston, TX) for 24 h (day 0), followed by RPMI/B27 minus insulin media for 48 h (day 1–3). Differentiating cells were treated with the Wnt signaling inhibitor IWP2 (5 μM; Tocris Bioscience, Bristol, United Kingdom) in RPMI/B27 minus insulin media for 48 h (day 3–5). At day 5 and day 7, culture media was replaced with fresh RPMI/B27 minus insulin media. From day 8, culture media was replaced with RPMI/B27 medium every 3 day up to day 20. Cells were incubated in a humidified incubator at 37 °C under 5% CO_2_.

### Purification of hESC-CMs

In the final phase of cardiomyocyte differentiation, cells were switched to glucose-free DMEM supplemented with Lactate (4 mM) on day 30 as described^39^. Differentiating hESCs were dissociated to single cells with 0.05% trypsin-EDTA at 37 °C for 5 min, collected with RPMI supplement with 20% FBS, and seeded on 0.1% gelatin-coated dish with RPMI supplement with B27.

### Flow cytometry

To prepare for single cells, hESCs were dissociated by Accutase treatment and hESC-CMs were dissociated with 0.05% trypsin-EDTA, followed by passing through 40 µm cell strainer. Cells were fixed with 4% paraformaldehyde for 20 min, washed with PBS, and permeabilized with 1% Triton-X-100 in PBS for 10 min. Cells were blocked with 5% BSA for 2 h and incubated with primary antibodies (TRA-1-60, TRA-1-81, SSEA4, and SSEA3 as hESC markers; αSA, cTnT, and MLC2a as cardiomyocyte markers) with 1:1000 dilution and overnight incubation at 4 °C. Secondary antibodies conjugated with fluorescent dyes were incubated with 1:2000 dilutions at 4 °C for 2 h. Fluorescence intensity of stained cells was measured using a CANTO II (BD Biosciences) and analyzed using FlowJo (ver 10, Tree Star Inc.).

### Reverse transcription-polymerase chain reaction (RT-PCR)

Total RNA was extracted from 80–90% confluent cultures using TRIzol reagent (Sigma) and reverse transcribed into cDNA using the Reverse Transcription cDNA Kit (#RT50KN; NanoHelix Co., Ltd). cDNA in 1 μL of the reaction mixture was amplified using the Ready-2 × -Go pre-mix PCR kit (#PMD008L; NanoHelix) and 10 pmol each of sense and antisense primers. The thermal cycle profile was as follows: denaturation at 95 °C for 30 s, annealing at 54 °C for 30 s depending on the primers used, and extension at 72 °C for 30 s. Each PCR reaction was carried out for 25–30 cycles. PCR products were analyzed by 1% agarose gel with ethidium bromide electrophoresis and photographed under UV trans-illumination. The primer sequences for RT-PCR are listed in the Supplementary Table S1.

### Quantitative RT-PCR (q-PCR)

Quantitative RT-PCR was performed using an ABI7500 (Applied Biosystems, Foster City, CA) sequence detection system, using SYBR Green PCR Master Mix (ABS-4309155, Applied Biosystems, Foster City, CA) according to the manufacturer’s instructions. Experiments were performed in duplicate, and the data were normalized to expression of GAPDH mRNA. Data were analyzed using the Δ (Δ CT) method and normalized to GAPDH. The primer sequences for q-PCR are listed in the Supplementary Table S2.

### Western blotting

Cells were washed twice with HBSS and then lysed in lysis buffer (Tris-HCl (20 mM), EGTA (1 mM), EDTA(1 mM), NaCl (10 mM), phenylmethylsulfonyl fluoride (0.1 mM), Na_3_VO_4_ (1 mM), sodium pyrophosphate (30 mM), β-glycerol phosphate (25 mM), 1% Triton X-100, pH 7.4). The cell lysates were centrifuged for 15 min at 4 °C, and the supernatants were used for western blotting. Lysates were resolved by sodium dodecyl sulfate-polyacrylamide gel electrophoresis, transferred onto nitrocellulose membranes, and then stained with 0.1% Ponceau S solution (Sigma, St Louis, MO, USA) to ensure equal loading of the samples. After being blocked with 5% non-fat milk for 30 min, the membranes were incubated with primary antibodies overnight, and the bound antibodies were visualized with horseradish peroxidase-conjugated secondary antibodies using the enhanced chemiluminescence Western blotting system (ECL, Amersham Biosciences, Piscataway, NJ, USA).

### Immunofluorescent staining

Undifferentiated hESCs and hESC-CMs were cultured on Matrigel-coated cover glasses. Cells were fixed with 4% paraformaldehyde for 20 min, washed with PBS, and permeabilized with 1% Triton-X-100 in PBS for 10 min. Cells were blocked with 5% BSA for 2 h and incubated with primary antibodies against hESC-specific markers (OCT4, SOX2, SSEA-4, TRA-1-81, TRA-1-60) or cardiomyocyte-specific markers (αSA, cTnT, MLC2v) with 1:200 dilution at 4 °C for overnight. Secondary antibodies conjugated with fluorescent dyes were stained with 1:500 dilutions at 4 °C for 2 h. Cells were mounted on the slide glass with Vectashield medium (Vector Laboratories) containing 4’,6-diamidino-2-phenylindole (DAPI) for nuclear staining. Images were collected with a confocal microscope system (Olympus FluoView FV1000, Olympus Corp., Tokyo, Japan).

### Electrophysiological analysis

Whole cell membrane currents were recorded using the whole-cell patch clamp technique with an EPC-8 amplifier (Medical System Corp., Darmstadt, Germany) and an NI-DAQ-7 digital interface (National Instruments, Union, CA). Patch pipettes were pulled from borosilicate capillaries (Clark Electromedical Intruments, Pangbourne, UK) using a PP-830 puller (Narishige Scientific Instrument Laboratory, Tokyo, Japan) to have a 2–3 MΩ when filled with pipette solution. The voltage signals were sampled at a rate of 1–3 kHz for K^+^ channel recordings, 4–6 kHz for Ca^2+^ channel recordings, and 15–10 kHz for Na^+^ channel recordings.

The extracellular solution for Na^+^ channel recordings contained: NaCl (130 mM), CsCl (15 mM), HEPES (5 mM), NaH_2_PO_4_ (0.33 mM), MgCl_2_ (0.5 mM), CaCl_2_ (1.8 mM), glucose 16.6, adjusted to pH 7.4 with NaOH. The internal solution for Na^+^ channel recordings contained: Cs-Aspartate (100 mM), CsCl (25 mM), HEPES (10 mM), EGTA (5 mM), NaCl (5.5 mM), Mg-ATP (4 mM), adjusted to pH 7.25 with CsOH. The extracellular solution for Ca^2+^ channel recordings contained: NaCl (135 mM), CsCl (10 mM), HEPES (5 mM), NaH_2_PO_4_ (0.33 mM), MgCl_2_ (0.5 mM), CaCl_2_ (1.8 mM), glucose (16.6 mM), adjusted to pH 7.4 with NaOH. The internal solution for Ca^2+^ channel recordings contained: CsCl (105 mM), TEA-Cl (22 mM), NaCl (5.5 mM), Mg-ATP(4 mM), EGTA (10 mM), HEPES (10 mM), adjusted to pH 7.25 with CsOH. The extracellular solution for K^+^ channel recordings contained: NaCl (137 mM), KCl (5.5 mM), HEPES (5 mM), glucose (15 mM), CaCl_2_ (1.7 mM), MgCl_2_ (0.7 mM), NaH_2_PO_4_ (0.33 mM), adjusted to pH 7.4 with NaOH. The internal solution for K^+^ channel recordings contained: K-aspartate (110 mM), KCl (27 mM), NaCl (5.5 mM), MgCl_2_ (1 mM), Mg-ATP (3 mM), HEPES (10 mM), EGTA (10 mM), adjusted to pH 7.2 with KOH. BIM (I) was purchased from Tocris Cookson (Ellisville, MO) and was dissolved in dissolved in dimethyl sulfoxide (DMSO).

Electrophysiological data were analyzed using The Origin 8.0 software (Microcal Software, Inc., Northampton, MA, USA). The Hill equation was used to obtain the half-maximal inhibitory (IC_50_) value and the Hill coefficient (*n*) as described below:

$$f=1{/}^{13},$$

where ƒ means the relative current inhibition (ƒ = 1 − *I*_drug_/*I*_control_) at the test potential, and [D] means the drug concentration.

### Statistical analysis

The results are presented as means ± S.E.M. Differences were tested for statistical significance using Student’s *t*-test, and a value of *p* < 0.05 was regarded significant.

## Electronic supplementary material

Supplementary Movie S1.

Supplementary Information
