# Supplementary material for: Functional expression and pharmaceutical efficacy of cardiac-specific ion channels in human embryonic stem cell-derived cardiomyocytes
Source: Sci Rep. 2017 Oct 23;7:13821. doi: 10.1038/s41598-017-14198-y (PMC5653792; doi:10.1038/s41598-017-14198-y)
Supplement: Supplementary file 2 — Supplementary Information [file 41598_2017_14198_MOESM2_ESM.pdf]

Functional expression and pharmaceutical efficacy of cardiac-specific ion channels in human embryonic stem cell-derived cardiomyocytes

Han Sol Kim[1#], Jung Won Yoon[2#], Hongliang Li[1], Geun Ok Jeong[2], Jin Ju Park[2], Sung Eun Shin[1], Il Ho Jang[3], Jae Ho Kim[2,4*], Won Sun Park[1*]

[1]Department of Physiology, Kangwon National University School of Medicine, Chuncheon, 24341, Republic of Korea; [2]Department of Physiology, School of Medicine, Pusan National University, Yangsan, 50612, Republic of Korea; [3]Department of Oral Biochemistry and Molecular Biology, Pusan National University School of Dentistry, Yangsan 50612, Republic of Korea; [4]Research Institute of Convergence Biomedical Science and Technology, Pusan National University Yangsan Hospital, Yangsan 50612, Republic of Korea

# These authors contributed equally to this work

*** Co-correspondence to:**

Jae Ho Kim, Ph.D., Department of Physiology, School of Medicine, Pusan National University, Yangsan 50612, Republic of Korea. Tel: +82-51-510-8073, Fax: +82-51-510-8076, E-mail: jhkimst@pusan.ac.kr

Won Sun Park, Ph.D., Department of Physiology, Kangwon National University School of Medicine, 1 Kangwondaehak-gil, Chuncheon, 24341, Republic of Korea.

Tel: +82-33-250-8834, Fax: +82-33-255-8809, E-mail: parkws@kangwon.ac.kr

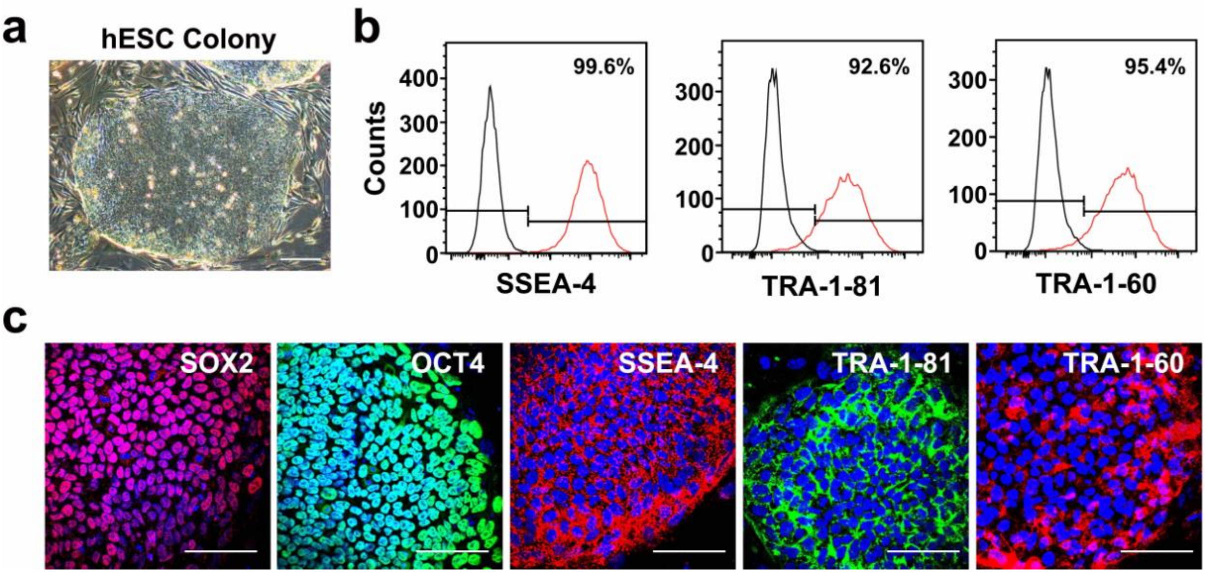

Supplementary Figure S1. Characterization of pluripotency in hESCs. (a) Phase bright image of hESC colony morphoplogy at day5. (b) Flowcytometry analysis of expression of SSEA-4, TRA-1-60, and TRA-1-81 was performed on hESCs. (c) Immunfluorescent staining for expression of SOX2(red), OCT4(green), SSEA-4(red), TRA-1-81(green), and TRA-1-60(red) was performed on undifferentiated hESCs. Nuclie were stained with DAPI and merge images are shown. Abbreviation; hESCs(human embryonic stem cells) (a) Scale bar=200μm. (c) Scale bar=100μm.

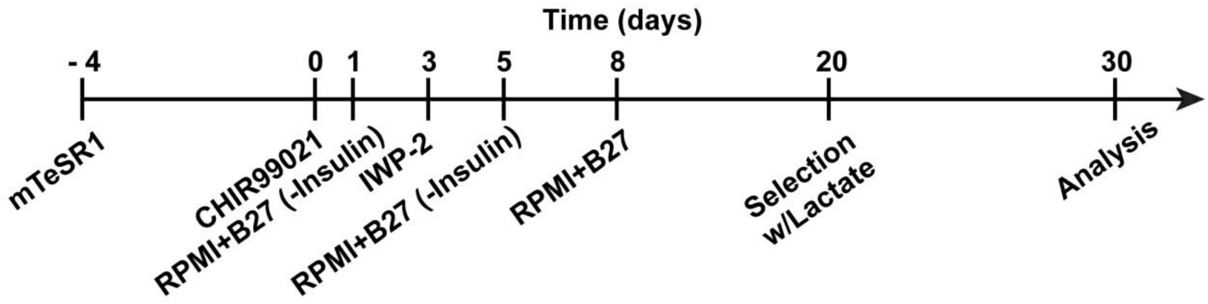

Supplementary Figure S2. Schematic presentation of differentiation protocol for H9 hESCs to cardiomyocytes. H9 cells were dissociated to single cell and cultured on Matrigel-coated plate at day -4.

Supplementary Table S1. The primer sequences for RT-PCR

Primer	Sequence
GATA4	F : CAG GCG TTG CAC AGA TAG TG<br>R : CCC GAC ACC CCA ATC TC
GATA6	F : TGT AGA GCC CAT CTT GAC CC<br>R : TCC CCC ACA ACA CAA CCT AC
cTnT	F : AAG ATC AGC TGA GGG AGA AG<br>R : GGT CTT GGA GAC TTT CTG GT
OCT4	F : ACG ACC ATC TGC CGC TTT GAG<br>R : GCC TCT CAC TCG GTT CTG AT
NANOG	F : CAA AGG CAA ACA ACC CAC TT<br>R :TCT GCT GGA GGC TGA GGT AT
$Ca_v1.1$	F : AAC GCC AAG AGG AGT ATT ATG<br>R : ATGGCTGTTGCTATGGTTGC
$Ca_v1.2$	F : CTG CAG GTG ATG ATG AGG TC<br>R : GCGGTGTTGTTGGCGTTGTT
$Ca_v3.1$	F : GAA GTG CTA CAG GGT GGA GGC<br>R : CCA GGT CTG CTG GGT CAG AGG
GAPDH	F : GGA GCC AAA AGG GTC ATC AT<br>R : GTG ATG GCA TGG ACT GTG GT

Supplementary Table S2. The primer sequences for Quantitative RT-PCR(q-PCR)

Primer	Sequence
α-MHC	F : TGT GGT GCC TCG TTC CA<br>R : TTT CGG AGG TAC TGG GCT G
β-MHC	F : GCA TTC TCC TGC TGT TTC CTT<br>R: TGG ATT CTC AAA CGT GTC TAG TGA
$Na_v1.5$	F : TGT ATG TCC TCA GTC CCT TC<br>R : CTT GAC CAG AGA CTC AAA GG
$K_v11.1$	F : AGT GAC CGT GAG ATC ATA GC<br>R : GAG TAG GGT GTG AAG ACA GC
GAPDH	F : GGA GCC AAA AGG GTC ATC AT<br>R : GTG ATG GCA TGG ACT GTG GT
